# Use of Non-Steroidal Anti-Inflammatory Drugs and Associated Gastroprotection in a Cohort of Workers

**DOI:** 10.3390/ijerph15091836

**Published:** 2018-08-24

**Authors:** María Jesús Lallana, Cristina Feja, Isabel Aguilar-Palacio, Sara Malo, María José Rabanaque

**Affiliations:** 1 Pharmacy Service in Primary Health Care, Aragones Health Service, 50009 Zaragoza, Spain; 2 Deparment of Microbiology, Preventive Medicine and Public Health, University of Zaragoza, 50009 Zaragoza, Spain; cfeja@unizar.es (C.F.); iaguilar@unizar.es (I.A.-P.); smalo@unizar.es (S.M.); rabanake@unizar.es (M.J.R.)

**Keywords:** non-steroidal anti-inflammatory drugs, gastrointestinal protection, pharmacoepidemiology

## Abstract

Background: This study describes the prevalence of non-steroidal anti-inflammatory drug (NSAID) use, and analyses prescribing patterns of NSAIDs and associated gastroprotection. Methods: The study population consisted of 5650 workers at the General Motors automobile assembly plant in Zaragoza, Spain. NSAID prescription data for 2014 were obtained from the prescription database of Aragon (Spain). NSAID consumption was determined based on the number of defined daily doses purchased per year. Heavy NSAIDs users were identified using Lorenz curves. Results: NSAID use in the cohort was high (40.7% of workers, 95% CI 39.4–41.9). The prescription of proton pump inhibitors increased with age. Gastrointestinal protection was lacking in some participants who were being treated with drugs associated with a high risk of gastrointestinal bleeding. Heavy NSAID users (defined as those above the 95th percentile of consumption), accounted for 26% of total DDDs, and consumed a greater proportion of coxibs than non-heavy users. Conclusions: The rate of NSAID consumption in the cohort was high. To reduce the risk of gastrointestinal complications, monitoring and adequate gastroprotection are essential in patients who are prescribed NSAIDs for long periods of time or who are treated concomitantly with drugs that increase the risk of gastrointestinal bleeding.

## 1. Introduction

Non-steroidal anti-inflammatory drugs (NSAIDs) are among the most commonly prescribed medications for inflammatory and musculoskeletal conditions [1,2]. Their mechanism of action consists of the inhibition of cyclooxygenase (COX) enzymes. This in turn impairs the synthesis and downstream effects of prostaglandins, prostacyclins, and thromboxanes. NSAIDs include traditional NSAIDs (e.g., ibuprofen, diclofenac, naproxen) and selective COX-2 inhibitors (coxibs). While NSAID treatment is associated with an increased risk of serious upper gastrointestinal (GI) events, this risk can be mitigated by prescribing a coxib rather than a non-selective NSAID [3,4].

The EPISER study [5] reported a rate of NSAID-related GI complications of 23.7%. Several epidemiological studies have attempted to estimate the risk of cardiovascular and GI events associated with NSAID use in general and with the use of individual NSAIDs [6,7]. The increased risk of gastrointestinal damage is common to all NSAIDs studied, is dose dependent, and is associated with long-term use [8]. Specifically, digestive adverse events should be monitored.

Gastropathy is one of the most important health problems caused by NSAIDs. Its clinical expression varies considerably, ranging from dyspepsia to serious complications that require hospitalisation (e.g., upper GI bleeding) [8,9]. The risk of NSAID-associated gastropathy can be increased by a variety of patient-related (e.g., age, history of peptic ulcer, smoking, alcohol consumption) and treatment-related (dose, duration, and type of NSAID used) factors, and by the concomitant use of other drugs including systemic corticosteroids, oral anticoagulants, platelet aggregation inhibitors, and other NSAIDs [10,11]. Where possible, physicians may prescribe lower doses of NSAIDs for short durations to reduce the risk of serious upper GI events, particularly in the subgroup of patients with the greatest background risk [8,10].

NSAID use is high [1], and is associated with the concomitant consumption of proton pump inhibitors (PPIs), which are frequently used to prevent NSAID-induced GI damage [12]. In this analysis of NSAID consumption in a cohort of workers, we analysed patterns of NSAID prescription and characterized the profile of heavy NSAID users and the consumption of PPIs by individuals prescribed NSAIDs.

## 2. Materials and Methods

The Aragon Workers’ Health Study (AWHS) is a prospective longitudinal study, with the aim of characterizing cardiometabolic factors in a population of middle-aged Mediterranean workers. We analysed data from routine annual health examinations of workers at a Spanish automobile assembly plant in Figueruelas, Zaragoza (Spain), who voluntarily agreed to participate in the study. The participants (*n* = 5650) were recruited between February 2009 and May 2010. Most of the workers in the cohort (86.5% of men and 60.5% of women) performed manual tasks. Active follow-up of participants is expected to continue through 2020. Further information on the AWHS can be found in Casasnovas et al. [13].

This observational descriptive study was designed to assess the prevalence of NSAID use in the AWHS cohort, and to characterise heavy NSAID users and the prescribing patterns of NSAIDs and associated gastroprotective agents in 2014. NSAID prescription data for AWHS participants was gathered from FarmaSalud, the prescription database of the government of Aragon, the Spanish autonomous community in which the assembly plant is located. This database collects data on all prescriptions dispensed at pharmacies in Aragon via the public health care system (i.e., prescribed by either a company physician or by general practitioners working in the public health system). FarmaSalud does not record prescriptions issued by private physicians or hospitals. Each record in the database corresponds to a prescription and contains the following information: an anonymous patient code, patient sex and birth date, dispensing date, Anatomical Therapeutic Chemical (ATC) code of the prescribed drug, number of defined daily doses (DDD), and number of packages dispensed. Drugs are classified according to the 2016 version of the World Health Organization ATC/DDD system [14]. Using an encrypted code provided by AWHS researchers, we identified prescriptions corresponding to NSAIDs (ATC code M01A).

Rates of dispensation per 100 workers and corresponding 95% confidence intervals (95% CI) were calculated. Rates were calculated as the number of individuals per 100 workers who filled at least one prescription for an NSAID in 2014. Data were further analysed after stratification for age and sex.

To assess the use of gastroprotective agents in conjunction with NSAIDs, we assessed the concomitant use of NSAIDs and proton pump inhibitors (PPI), which is the class of drug most commonly used to prevent NSAID-induced gastropathy in Spain [12]. Patients were categorised as users of gastroprotective agents if they filled a prescription for a PPI (ATC code A02BC) within two months of filling the first NSAID prescription (set as index date). Among workers treated with NSAIDs, the concomitant use of other drugs that increase the risk of GI damage was also analysed. The drugs included in this analysis were platelet aggregation inhibitors (B01AC), anticoagulants (B01AA, B01AB, B01AE, B01AF), corticosteroids (H02, with the exception of topically or locally applied corticosteroids), and selective serotonin-reuptake inhibitors (SSRIs, ATC code N06AB). Concomitantly prescribed drugs were defined as those prescribed within two months of the NSAID prescription. A χ^2^ test was used to compare the use of gastroprotective agents. Statistical significance was set at *p* < 0.05.

The annual amount of DDDs was used to categorise the NSAID consumption. We set the limit for low use to 30 DDDs and chronic use to more than 90 DDDs. Lorenz curves were used to identify heavy consumers of NSAIDs within the cohort. This analytical tool was applied to NSAID use to highlight the unequal distribution of drug consumption within the study population [15,16]. In the Lorenz curve, the *x*-axis represents the accumulated percentage of drug users and the *y*-axis the accumulated percentage of total consumption (DDD) per year. Thus, it shows the proportion of drug use accounted for by percentiles of drugs users, ranked according to their volume of consumption, measured in DDD. We defined heavy users of NSAIDs as those above the 95th percentile of consumption (i.e., the 5% of patients with the highest DDD values). Within this group, distribution by age and prescribing patterns per pharmacological subgroup were compared with those observed for the remainder of NSAID consumers (non-heavy users). The two groups were compared using a χ^2^ test, with statistical significance set at *p* < 0.05.

## 3. Ethics

Participants in the AWHS provided written informed consent on enrolment. The present study was approved by the Clinical Research Ethics Committee of Aragon (RAB-EST-2014-01. FIS13/1668. Approved February 2014).

## 4. Results

Of the 5650 participants in the AWHS, 2294 (40.7%) filled at least one prescription for an NSAID in 2014. Analysis of the rate of prescription of NSAIDs by age group and sex (Table 1) revealed a higher rate in older age groups. In men, the rate for those under 50 years of age was the lowest rate: 32.9% (95% CI 30.3–35.5), while the highest rate was observed for those over 59 years of age (46.7%, 95% CI 43.7–49.7). For women, the total rate of dispensation of NSAIDs was 38.4%. NSAID consumption in this group also increased with age, with higher rates observed in the oldest age group (over 55 years).

The most commonly prescribed NSAID subgroup was non-selective NSAIDs: prescriptions for drugs corresponding to the ATC codes M01AE (propionic acid derivatives) and M01AB (acetic acid derivatives) were filled by 69.9% and 28.9% of workers, respectively. The next most prescribed subgroups were M01AX that includes symptomatic slow-acting drugs for osteoarthritis (SYSADOAs) and M01AH (selective cyclooxygenase-2 inhibitors or coxibs). Ibuprofen was the most frequently prescribed NSAID (48.2% of workers treated with NSAIDs were prescribed this drug). The next most commonly prescribed traditional NSAIDs were diclofenac and dexketoprofen. Etoricoxib was prescribed more frequently than celecoxib, while chondroitin sulfate was the most prescribed drug of the M01AX subgroup.

Of the participants who were prescribed NSAIDs and PPIs, 71.7% were over 55 years of age. In the group of workers that used NSAIDs without gastroprotection, 311 (22.5%) were over 59 years of age. The prescription of gastroprotective agents was significantly higher in patients who were being treated with NSAIDs and platelet aggregation inhibitors, anticoagulants, or corticosteroids, although not all workers using these drugs associated with a high risk of GI events had been prescribed a PPI (Table 2).

NSAID: Non-steroidal anti-inflammatory drugs. PPI: Proton Pump Inhibitors. SSRI: Selective Serotonin-Reuptake Inhibitor. Anticoagulants (Vitamin K antagonists 13 patients; Heparins: 24 patients; Direct factor Xa inhibitors: three patients). People treated with an NSAID and PPI filled a prescription for a PPI within two months of filling the first NSAID prescription (Attached to Table 2).

**Figure 1:**
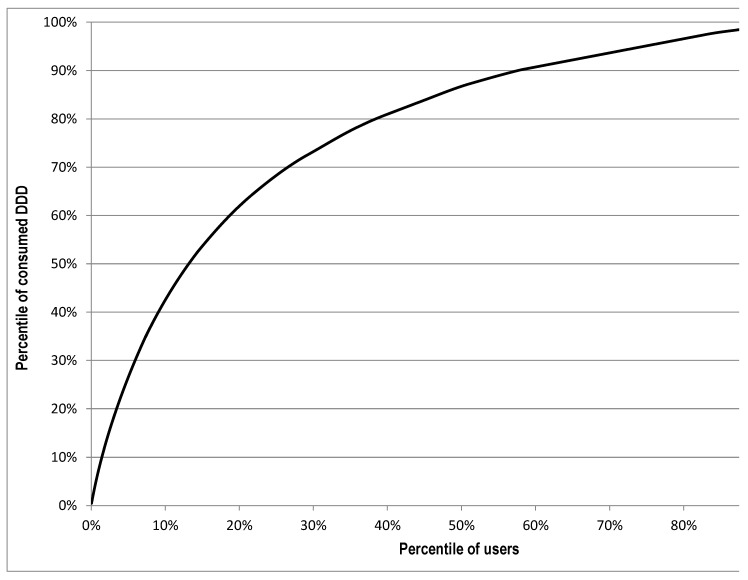
Lorenz curve of non steroidal anti-inflammatory drugs use in the cohort of workers, 2014.

The total consumption of NSAIDs in the cohort was 76 DDD/1000 inhabitants per day (DID) in 2014. In total, 1130 workers were prescribed <31 DDD of NSAIDs, while 484 (21.1%) were prescribed >90 DDD. The Lorenz curve (Figure 1) deviated to the extreme upper left, indicating that a small number of workers (heavy users) accounted for a substantial portion of the total number of DDDs. Heavy NSAID users in our study, defined as those above the 95th percentile of consumption, were responsible for 26.6% of the total number of DDDs in the cohort. The mean consumption of NSAIDs within this group was 362 DDD/year.

Rates of prescription of coxibs (14.2% vs. 6.8%, *p* < 0.001) and SYSADOAs (37.8% vs. 16.3%, *p* < 0.001) among heavy NSAID users were significantly higher than those observed for non-heavy users (Table 3).

## 5. Discussion

NSAIDs were widely used in our cohort: 40.7% of workers received an NSAID prescription in 2014. This rate is considerably higher than the rates reported in other studies of the general population; a Finnish study conducted in 2000 reported a prevalence of 17.1% [17], while a 2005 study in Denmark reported a rate of 16.9% [18]. In Spain, a study carried out in Navarra found that 14% of the general population had been treated with an NSAID during the first three months of 2016 [19]. It should be borne in mind that our cohort mainly consisted of younger individuals, most of whom were men. A high rate of NSAID use would not be expected in this group; the subgroups with the highest rates of NSAID use are the elderly, women, and patients with rheumatic diseases [17,18]. However, studies have shown a strong correlation between the prevalence of musculoskeletal disorders and NSAID use [17,19]. As such, the fact that all participants in the present study were automotive industry workers, in whom the prevalence of musculoskeletal disorders that require NSAID treatment may be increased [20], may explain the higher than expected rate of NSAID prescription observed.

Using the DDD per 1000 inhabitants per day (DID) as the unit of measure, NSAID consumption in the cohort was 76 DID, which is significantly higher than that reported in Spain in 2012 (49 DID) [2] or in Navarra in 2016 (<40 DID) [19]. However, the results of the analyses of the most commonly prescribed NSAIDs (ibuprofen and related compounds [M01AE]) were in agreement with those reported in previous studies [1,17,18,19].

Several studies have shown that the use of NSAIDs is associated with many adverse effects and hospitalizations due to GI complications [6,21]. According to a study by Laporte and coworkers [22], 38% of all cases of GI haemorrhage were attributable to the use of NSAIDs (152 cases per million inhabitants/year). Factors that increase NSAID-associated toxicity include age, a previous history of peptic ulcers, and the concomitant use of other drugs, including other NSAIDs, oral anticoagulants, platelet aggregation inhibitors (including low-dose aspirin), and corticosteroids [10].

In our cohort, 311 patients over 59 years of age were prescribed an NSAID without a gastroprotective agent. A recent study that analysed hospital admissions for NSAID-induced gastropathy found that the most common risk factors for these admissions were age and concomitant treatments with other drugs that enhance the GI toxicity of NSAIDs. The same study found that a large proportion of patients who met the criteria for gastroprotection were not prescribed gastroprotective drugs [21].

In agreement with previous findings [2,19,23], the most commonly used NSAIDs in the cohort were ibuprofen and diclofenac, which are associated with a low-to-intermediate risk of GI complications, depending on the doses used [24,25,26]. COX-2 inhibitors and SYSADOAs also have a lower GI risk profile. Our analyses revealed the possible underutilization of gastroprotective agents in individuals of over 60 years of age and in patients treated concomitantly with drugs that increase the risk of bleeding (e.g., platelet aggregation inhibitors and anticoagulants). Although other studies have reported inadequate gastroprotective treatment in patients treated with NSAIDs, either due to over- or underutilization [21,23,27,28], patients treated with NSAIDs and concomitant platelet aggregation inhibitors usually receive gastroprotection [23]. However, the rate of use of gastroprotective agents is lower in patients treated with low-dose acetylsalicylic acid [2], for which there appears to be a lower perception of risk on the part of medical professionals. There is considerable variability in the literature regarding the use of gastroprotective agents in patients receiving concomitant NSAIDs and an SSRI agent: some studies have reported no increased risk of bleeding [6,29], which may explain no differences in the use of gastroprotective agents in these patients in the cohort.

Methods used to detect high levels of drug consumption usually focus on patients who consume the highest number of DDDs per year [16,17,27]. In our cohort, NSAID use increased with age. Moreover, almost 75% of heavy users were 55 or older. Other studies have reported a high prevalence of heavy NSAID use among elderly patients [17,27]. We found that among patients treated with NSAIDs, the prescription profile of heavy users differed to that of other users: a larger proportion of the former group were prescribed SYSADOAs and coxibs, while traditional NSAIDs were more commonly prescribed in the latter group.

In our cohort, chondroitin sulfate was the most commonly prescribed SYSADOA, particularly among heavy users of NSAIDs, despite a lack of solid evidence supporting its efficacy [30]. The latest revision of the NICE guide does not recommend the use of chondroitin sulfate in osteoarthritis patients [31], despite its good safety profile.

High doses and/or long treatment durations may account for the high levels of NSAID consumption observed in the heavy user subgroup. Both these scenarios are associated with an increased risk of GI toxicity [6,8,24,27]. We found that heavy users were prescribed an average of 362 DDDs of an NSAID per year, implying a consumption of one standard dose per day for the entire year by participants in this group. Almost 75% of heavy users were older than 55 years. Both age and heavy use of NSAIDs are risk factors of gastrointestinal damage. In these situations, GPs are prone to select coxib or SYSADOA because these drugs have a low risk of gastrointestinal toxicity [6].

The main limitation of this study relates to the source of data used. In analysing the number of NSAID prescriptions dispensed at pharmacies in Aragon, we may have underestimated NSAID consumption in this population, since these drugs can be obtained without prescription. As such, the true rate of NSAID use may be higher than that reported here. Furthermore, because the prescription data available in the FarmaSalud database does not include clinical variables, it was not possible to determine the appropriateness of the treatments prescribed. This is an important factor to consider when assessing the relevance of gastroprotection in this group. Another limitation relates to the study population: since this cohort worked exclusively in the automotive sector, and the study population is largely composed of men, reflecting the sex distribution in the factory, the results obtained could present some differences with the general population.

Baseline data from the AWHS cohort show a high prevalence of cardiovascular risk factors [13]. Follow-up of this cohort will allow the assessment of cardiovascular events and study the relation with the drugs consumed, as NSAIDs.

## 6. Conclusions

Based on our findings, we can conclude that the use of NSAIDs in this cohort of workers is much higher than that found in the general population, probably due to the greater prevalence of musculoskeletal pathologies associated with the specific work activities of the cohort. The cohort included a group of patients who were prescribed NSAIDs for very long periods of time, which may increase the risk of adverse effects associated with these drugs. Exposure of patients to long term use of NSAIDs should be reduced by regular analysis of a patient’s pain and a consideration of other treatment options.

Physicians should carefully weigh the risks and benefits of prescribing NSAIDs in patients at a high risk of gastrointestinal damage: gastroprotection with PPI is necessary when dealing with older people or people treated with drugs that increases serious gastrointestinal events.

## Figures and Tables

**Table 1:** Rate of dispensation of non-steroidal anti-inflammatories stratificated for age and sex in the cohort of workers in 2014.

	Age Groups (Years)				
	<50 (95% CI)	50–54 (95% CI)	55–59 (95% CI)	>59 (95% CI)	Total (95% CI)
Male	32.9 (30.3–35.5)	41.9 (39.0–44.8)	43.0 (40.7–45.3)	46.7 (43.7–49.7)	40.8 (39.5–42.2)
Female	32.1 (25.7–38.4)	40.8 (29.4–52.3)	48.5 (36.6–60.4)	53.3 (35.5–71.2)	38.4 (33.5–43.3)
TOTAL	32.8 (30.4–35.2)	41.8 (39.0–44.7)	43.2 (40.9–45.4)	46.9 (43.9–49.8)	40.7 (39.4–42.0)

Number of workers in the cohort: 5650 (Male: 5268; Female: 382). 95% CI: 95% confidence intervals. Rate per 100 workers.

**Table 2:** Characteristics of individuals treated with non-steroidal anti-inflammatories (NSAID) or NSAID and proton pump inhibitors and concomitant drugs at a high risk of gastrointestinal events.

	People Treated with NSAID without PPI	People Treated with NSAID and PPI	*p*-Value
**Number of individuals**	1381	690	
**Gender, *n* (%)**			
Male	1278 (92.5)	665 (96.4)	
Female	103 (7.5)	25 (3.6)	
**Age groups, *n* (%)**			
<50 years	320 (23.2)	82 (11.9)	<0.001
50–54 years	268 (19.4)	113 (16.4)	
55–59 years	482 (34.9)	273 (39.5)	
>59 years	311 (22.5)	222 (32.2)	
**Concomitant medical therapy, *n* (%)**			
Anticoagulant drugs	15 (1.1)	25 (3.6)	<0.001
Platelet aggregation inhibitors	47 (3.4)	78 (11.3)	<0.001
Corticosteroids	17 (1.2)	23 (3.3)	0.001
SSRI	33 (2.4)	24 (3.5)	0.150

**Table 3:** Characterization of heavy users and non-heavy users of non-steroidal anti-inflammatory drugs.

	Heavy Users of NSAID	Non-Heavy Users of NSAID	*p*-Value
**Number of individuals**	115	2175	
**Gender, *n* (%)**			
Male	111	2034	
Female	4	141	
**Age group, *n* (%)**			
<50 years	6 (5.2)	478 (22.0)	<0.001
50–54 years	23 (20.0)	468 (21.5)	
55–59 years	40 (34.8)	767 (35.3)	
>59 years	46 (40.0)	462 (21.2)	
**NSAID subgroup, *n* (% of total prescriptions)**			
M01AB Acetic acid derivatives and related substances	249 (15.1)	1102 (18.7)	<0.001
M01AE Propionic acid derivatives	511 (30.9)	3342 (56.7)	<0.001
M01AH Selective COX-2 inhibitors (coxibs)	234 (14.2)	396 (6.7)	<0.001
M01AX Other anti-inflammatory and antirheumatic agents	624 (37.8)	968 (16.4)	<0.001
Others (oxicams and fenamates)	33 (2.0)	82 (1.4)	<0.001
Total prescriptions	1651	5890	
