# Addendum: Lallana, M.J. et al. Use of Non-Steroidal Anti-Inflammatory Drugs and Associated Gastroprotection in a Cohort of Workers. 2018, *15*, 1836

**DOI:** 10.3390/ijerph17238997

**Published:** 2020-12-03

**Authors:** María Jesús Lallana, Cristina Feja, Isabel Aguilar-Palacio, Sara Malo, María José Rabanaque

**Affiliations:** 1 Pharmacy Service in Primary Health Care, Aragones Health Service, 50009 Zaragoza, Spain; 2 Department of Microbiology, Preventive Medicine and Public Health, University of Zaragoza, 50009 Zaragoza, Spain; cfeja@aragon.es (C.F.); iaguilar@unizar.es (I.A.-P.); smalo@unizar.es (S.M.); rabanake@unizar.es (M.J.R.)

The authors wish to make the following correction to this paper [1]:

## Change in Funding

In the original version of our article (Lallana, M.J. et al. Use of Non-Steroidal Anti-Inflammatory Drugs and Associated Gastroprotection in a Cohort of Workers. 2018, 15, 1836), insufficient source of funding was given. The authors wish to change the information in the Funding section from:

**Funding:** This study was funded by the Instituto Carlos III, grant number (PI17/01704).

to the correct version as follows:

**Funding:** This study was funded by Proyecto del Fondo de Investigación Sanitaria, Instituto de Salud Carlos III (Ministerio de Ciencia e Innovación) and Fondo Europeo de Desarrollo Regional (FEDER)(PI17/011704).

The authors would like to apologize for any inconvenience caused to the readers by these changes.
